# Genetic Diversity and Temporal Shifts of Porcine Reproductive and Respiratory Syndrome Virus Type 2 (PRRSV-2) Strains in Japan (2020–2023): Evidence of Modified Live Vaccine Influence on Cluster Distribution

**DOI:** 10.3390/epidemiologia6040077

**Published:** 2025-11-06

**Authors:** Yoriko Yonezawa, Osamu Taira, Atsushi Kato, Ryosuke Takai, Ryohei Nukui, Nobuyuki Tsutsumi, Ryota Matsuyama, Kohei Makita

**Affiliations:** 1 Nisseiken Co., Ltd., 9-2221-1 Shin-machi, Ome 198-0024, Tokyo, Japan; y.yonezawa@jp-nisseiken.co.jp (Y.Y.); r.nukui@jp-nisseiken.co.jp (R.N.); tsutsumi@nibs.or.jp (N.T.); 2 Veterinary Epidemiology Unit, Graduate School of Veterinary Medicine, Rakuno Gakuen University, 582 Bunkyodai Midorimachi, Ebetsu 069-8501, Hokkaido, Japan; 3 Nippon Institute for Biological Science, 9-2221-1 Shin-machi, Ome 198-0024, Tokyo, Japan; o.taira@nibs.or.jp (O.T.); r.takai@nibs.or.jp (R.T.); 4 National Institute of Animal Health, Tsukuba 305-0856, Ibaraki, Japan

**Keywords:** porcine reproductive and respiratory syndrome virus 2, PRRSV-2, phylogenetic analysis, modified live vaccine, MLV

## Abstract

Background: Porcine reproductive and respiratory syndrome virus type 2 (PRRSV-2) remains a significant threat to swine production globally, including Japan. While the genetic diversity of PRRSV-2 has been reported previously, the potential association with modified live vaccines (MLVs) is not well understood. This study aimed to characterize PRRSV-2 strains currently circulating in Japan and assess possible links with MLVs. Methods: A total of 1190-nucleotide open reading frame 5 sequences of PRRSV-2 were collected across Japan between 2020 and 2023, and phylogenetic analyses were performed to classify genetic clusters. Additionally, correlations between cluster distribution and MLV usage were examined, using sequences detected in the Kanto region. Results: Phylogenetic analysis revealed that 48.5% of the sequences belonged to Cluster III, with a median nucleotide identity of 88.2% to the Japanese reference strain EDRD-1. Notably, the sequence identity between the strains detected in this study and EDRD-1 was significantly lower than that of strains identified in 1992–1993 (*p* < 0.05). In the Kanto region, Cluster I and II variants, which exhibited high sequence homology to MLV strains, were exclusively detected on farms with a history of MLV usage. Furthermore, Cluster IV displayed substantial genetic divergence, suggesting it comprises a heterogeneous group of distinct lineages. Conclusions: These findings demonstrated the temporal changes in the genetic diversity of Cluster III and provided suggestions of a possible influence that MLV usage influences PRRSV-2 cluster distribution, with Clusters I and II likely representing vaccine-origin viruses. The marked heterogeneity of Cluster IV also highlights the limitations of the current cluster-based classification.

## 1. Introduction

Porcine reproductive and respiratory syndrome (PRRS) caused by PRRS virus (PRRSV) is a contagious disease causing reproductive failure in sows and respiratory distress in piglets [1]. First identified in the late 1980s in the U.S. and Europe [2], PRRSV has since led to significant economic losses globally, including an estimated over 1.2 billion USD annually in the U.S. during 2016 to 2020 [3]. PRRSV, a single-stranded RNA virus belonging to *Betaarterivirus* genus, *Arteriviridae* family, consists of two species: *Betaarterivirus europensis* (PRRSV-1, formerly European genotype) and *Betaarterivirus americense* (PRRSV-2, formerly American genotype) [4]. The virus exhibits frequent mutations, particularly in the open reading frame (ORF) 5 gene, which encodes glycoprotein (GP) 5—a key membrane protein involved in host cell binding and virus neutralization [5,6]. ORF5 sequence analysis is widely used to classify PRRSV strains and study their genetic evolution [7,8,9,10,11].

In Japan, both PRRSV-1 and PRRSV-2 have been reported; however, PRRSV-2 is the predominant genotype currently circulating nationwide and is responsible for the majority of PRRS-related clinical cases and genetic diversity observed in domestic swine herds [12,13]. As of 2024, Japan had approximately 3130 pig farms, with a total pig population of 8.8 million, of which 758,300 were breeding sows [14]. A nationwide survey conducted in 2010 reported that over 80% of farms tested positive for PRRSV, and that infected farms experienced increased mortality and reduced growth performance [15]. Additionally, PRRSV-related economic losses in Japan were estimated to exceed 28 billion Japanese yen annually between 2006 and 2008 [16].

The classification system for PRRSV-2 has evolved over time. Initially, phylogenetic typing of PRRSV-2 employed restriction fragment length polymorphism (RFLP) typing based on restriction enzyme (MluI, HincII, SacII) cleavage patterns of the DNA fragment coding for the ORF5 gene in 1998 to discriminate RespPRRSV/Ingelvac PRRS MLV strains (RFLP 2-5-2) from wild-type strains [17]. Although this technique was widely used in North America, it presented limitations in assessing the genetic similarity of different strains [8,18]. Subsequently, a sequence-based phylogeny of ORF5 was established in 2010 [19], enabling a more detailed understanding of the genetic divergence of PRRSV-2. Through this approach, PRRSV-2 has been classified into 11 lineages and 21 sub-lineages [8]. This lineage taxonomy has gained wide acceptance internationally, and global data revealed that lineage distribution exhibits regional variation, with predominant lineages replaced over time [20,21]. On the other hand, in Japan, the classification of PRRSV-2 was reported by Yoshii et al. in 2005 to form five clusters [22], which subsequently became the standard system for the genotyping of PRRSV-2 in Japan. Historical analyses reveal that only Clusters I and III were detected in 1992–1993 [22], whereas Clusters II and V, in addition to Clusters I and III, were identified in 2000–2001 [23]. Furthermore, comparative sequence analyses of isolates collected between 1992 and 2008 demonstrated a progressive increase in nucleotide diversity within Cluster III, indicating ongoing genomic evolution over time [23]. Notably, Cluster IV was detected for the first time in Japan in 2008 [23]. More recent data indicate that cluster II predominated among the sequences detected in 2018–2020, followed by Cluster IV, while Cluster III, which had previously dominated, has exhibited a decline [13].

Control strategies for PRRS include a variety of approaches, namely early detection and isolation, and hygiene control, with vaccination representing one of the most crucial and widely implemented interventions worldwide [24,25,26]. The commercial PRRSV vaccines are dominated by two types, modified live vaccine (MLV) and killed vaccine, with MLV being more extensively used [24,27]. In the Japanese market, MLV products include Ingelvac PRRS^®^ MLV, Fostera^®^ PRRS, and Unistrain^®^ PRRS from three different pharmaceutical companies. Ingelvac PRRS^®^ MLV was launched in 1998, and Fostera^®^ PRRS was launched in 2018. The active ingredient of the vaccines corresponds to Clusters I and II of PRRSV-2, respectively. Notably, only Unistrain^®^ PRRS uses PRRSV-1 as a vaccine strain and was launched in 2023.

The extensive genetic variability of PRRSV represents one of the main challenges to the effective control of the virus [20,28,29]. Persistent PRRSV gene changes over time have been reported in PRRSV-endemic areas, and the accumulation of these changes is expected to impair the effectiveness of vaccination-based disease control strategies as they produce genetic and antigenic diversity [20,29]. Therefore, a comprehensive phylogenetic analysis of PRRSV is critical for understanding the evolutionary dynamics of the virus and guiding the development of more effective vaccination and control measures.

Given these considerations, this study firstly aimed to analyze the genetic divergence of PRRSV-2 detected in Japan during 2020–2023 and to elucidate temporal changes in the genetic diversity of PRRSV in the country by comparing these contemporary sequences with those published in previous studies. Secondly, the proportions of the farms using MLV were compared between clusters to assess the relationship between MLV use and the distribution of clusters to which prevalent strains belong.

## 2. Materials and Methods

### 2.1. Study 1. Description of Genetic Variability of PRRSV-2 Detected in Japan During 2020–2023

#### 2.1.1. Study 1.1: Broad Regional Analysis (2020–2023)

##### Sample Collection for Study 1.1

Between 2020 and 2023, samples were collected from 69 farms across 18 prefectures as part of health screening and disease investigation. A total of 1190 sequences were obtained from 1136 serum samples, 37 oral fluid samples, and 17 organ samples. Detailed information regarding the clinical status of individual animals and the disease history of the farms from which many of the samples originated was not consistently available.

##### Sample Analysis for Study 1.1

All laboratory procedures—including RNA extraction, qualitative and quantitative reverse transcription polymerase chain reaction (RT-PCR/RT-qPCR) targeting PRRSV-2, and sequencing of the ORF5 region—were performed by SMC Co., Ltd. (Kanagawa, Japan) or Nippon Institute for Biological Science (NIBS) (Toyo, Japan). Sequences were aligned in MUSCLE (University of Colorado, Boulder, CO, USA) using default settings. Phylogenetic analyses were conducted using the Neighbor-joining method, following protocols established in previous studies [13,22,23], and implemented in MEGA version 11 software (Center for Evolutionary Medicine and Informatics, Tempe, AZ, USA) [30]. Bootstrap analysis was performed with 1000 replications. The phylogenetic analyses were conducted using previously published strain sequences and strains isolated by NIBS between 1997 and 2019. Table 1 shows strains that were isolated domestically, while Table 2 shows strains that were isolated overseas [13,22,23]. Sequences were clustered based on phylogenetic analysis following a cluster classification scheme described in previous reports [13,22,23]. To facilitate the cluster classification, sequences were grouped by farm. For each farm, a phylogenetic tree was constructed together with reference strains, and when multiple sequences from the same farm clustered on the same branch, only one representative sequence was retained. As a result, one to four representative sequences per farm were selected and included in the phylogenetic tree. In total, 97 representative sequences were selected and incorporated into the final phylogenetic analysis. To evaluate the diversity of each cluster, 1190 sequences were compared to the earliest reference strain within each cluster, and nucleotide homology was calculated. Within-cluster homology was also calculated and reported as mean or median values with interquartile ranges (IQR) to assess genetic diversity. For clusters I and II, which include MLV strains, homology was also calculated between sequences belonging to each cluster and the respective MLV strains. Sequences in Cluster I were compared with previously published sequences detected in Japan between 1992 and 2008 [22,23], prior to the introduction of the Fostera^®^ PRRS vaccine. For Cluster III, homology was assessed against sequences detected during 1992–1993, following previous reports in which EDRD-1 was used as the reference strain. Additionally, the sequences obtained between 2020 and 2023 were subjected to lineage classification using ISU PRRSView (Iowa State University, Ames, IA, USA), hosted by Iowa State University [20,21,31].

#### 2.1.2. Study 1.2: Regional Analysis of the Kanto Region (2020–2023)

##### Sample Collection for Study 1.2

Study 1.2 focused on sequences derived from samples collected in the Kanto region, which represent a subset of the nationwide dataset described in Study 1.1. Specifically, 576 sequences were obtained from samples that tested positive for PRRSV-2 by RT-PCR or RT-qPCR, out of a total of 1670 samples submitted for health screening or disease investigation, comprising 1275 serum samples, 380 oral fluid samples, and 15 organ samples. These samples were collected from 34 farms across four prefectures in the Kanto region: Chiba (18 farms), Ibaraki (10 farms), Tochigi (5 farms), and Kanagawa (1 farm).

##### Sample Analysis for Study 1.2

A total of 1670 samples were subjected to RT-qPCR or RT-PCR targeting PRRSV-2. For each RT-PCR-positive batch, one representative sample was selected for sequence analysis. All laboratory procedures—including RNA extraction, RT-PCR/RT-qPCR, and sequencing of the ORF5 region—were performed by SMC Co., Ltd. or NIBS, following the same protocols as in Study 1.1. All 576 sequences obtained in Study 1.2 were already included in the dataset analyzed in Study 1.1. and had been classified according to its established cluster framework.

#### 2.1.3. Statistical Analysis for Study 1

A descriptive epizootiological analysis was carried out to investigate the geographical and production-stage distribution of PRRSV-2 clusters. The Mann–Whitney U test was used to compare the sequence identity of strains belonging to the same cluster between this study and previous studies.

### 2.2. Study 2. Assessment of the Relationship Between MLV Use and the Distribution of PRRSV-2 Clusters of Prevalent Strains

#### 2.2.1. Sample Collection and Sequencing for Study 2

To assess the relationship between the use of MLV and the distribution of prevalent strains, a subset of 576 sequences derived from the Kanto region in study 1.2 was analyzed. In addition, information regarding the use of MLV was collected from all participating farms.

#### 2.2.2. Statistical Analysis for Study 2

Chi-square tests were used to compare the proportions of farms using MLV among the clusters, including only farms with known MLV usage status. As four clusters, I–IV, except Cluster V, were identified, pairwise comparisons of proportions were subsequently conducted using the pairwise.prop.test function in R version 4.3.3. [32] and RStudio Cloud (https://www.posit.co/) [33].

## 3. Results

### 3.1. Study 1: Description of Genetic Variability of PRRSV-2 Detected in Japan During 2020–2023 and Comparison of Sequence Homology with Previous Reports

#### 3.1.1. Broad Regional Phylogenetic Analysis and Cluster Identification (Study 1.1)

**Figure 1:**
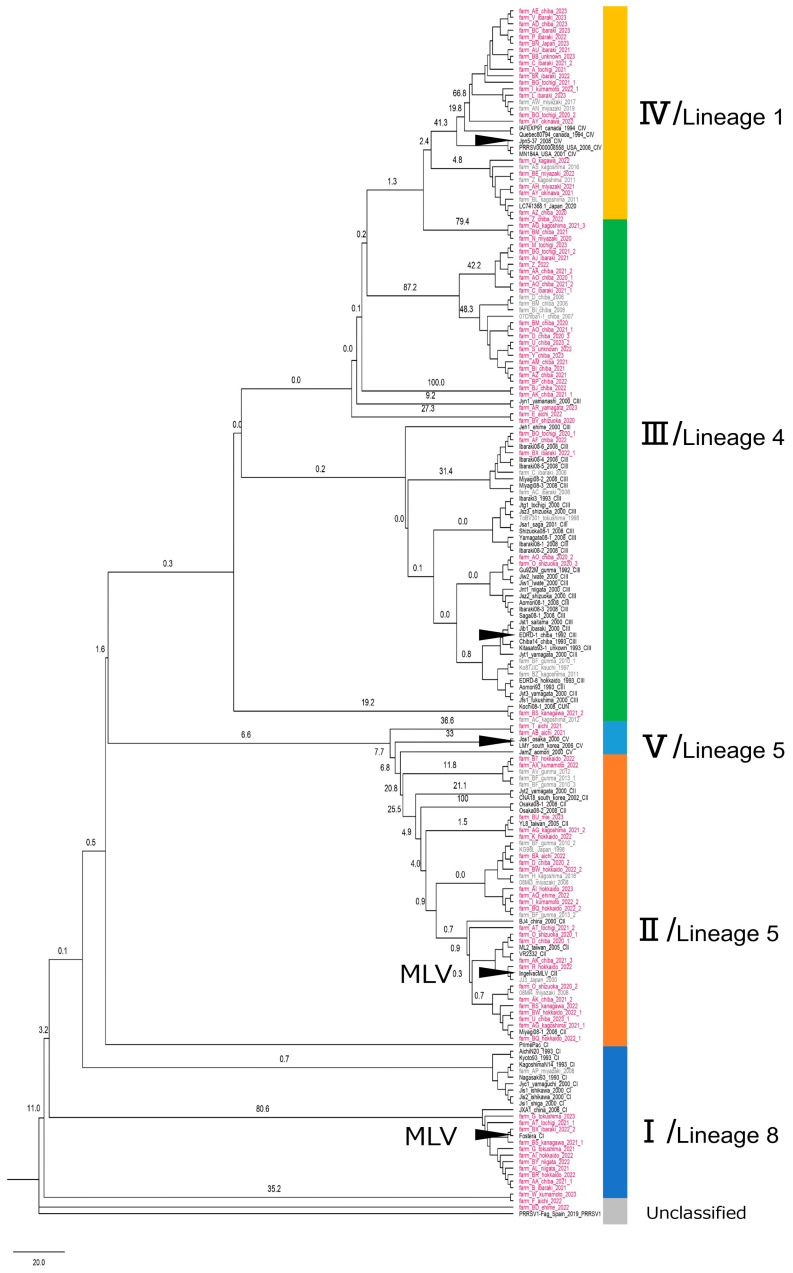
Phylogenetic tree and cluster classification of PRRSV strains detected between 1992 and 2023 based on reference sequences. Clusters are color-coded as follows: Cluster I (blue), Cluster II (orange), Cluster III (gray), Cluster IV (yellow), Cluster V (light blue), and Unclassified (green). Black labels indicate the reference strains, gray labels indicate strains collected between 1997 and 2019 at NIBS, and red labels denote strains analyzed in the present study. Arrowheads indicate the reference strains used for the homology analysis. Sequences were grouped by branch, and one to three representative sequences were selected per farm. In total, 97 representative sequences obtained between 2020 and 2023 were included in the phylogenetic tree. Bootstrap analysis was performed with 1000 replications.

PRRSV-2 ORF5 gene sequences collected between 2020 and 2023 were classified into five clusters, I–V, based on reference sequences reported in previous studies [13,22,23]. Sequences that did not correspond to any established cluster were designated as Unclassified (Figure 1). Cluster I included numerous sequences closely related to the MLV vaccine strain, Fostera^®^ PRRS. Among the sequences assigned to Cluster I, those detected in this study (2020–2023) and reference strains detected between 1993 and 2008 in Japan formed a separate grouping on the phylogenetic tree. Similarly, Cluster II contained a large number of sequences, many of which exhibited close homology to the MLV vaccine strain, Ingelvac^®^ PRRS MLV. Cluster III showed marked genetic divergence; sequences detected between 2020 and 2023 primarily occupied phylogenetic positions distinct from those identified between 1992 and 2008. The phylogenetic tree also revealed extensive branching within Cluster IV. The proportions of PRRSV-2 ORF5 gene sequences assigned to each cluster were calculated based on the total number of sequences obtained (n = 1190). Specifically, 13.3% for Cluster I, 18.6% for Cluster II, 48.5% for Cluster III, 18.4% for Cluster IV, 0.2% for Cluster V, and 1.0% for Unclassified. Regarding the distribution of clusters at the pig farm level, 53 farms (76.8%) harbored PRRSV-2 strains belonging to only one cluster, 15 farms (21.7%) contained those belonging to two clusters, and 1 farm (1.4%) hosted those classified into three clusters. According to the lineage classification, all sequences assigned to Cluster I were identified as lineage 8 (L8C). In Cluster II, all sequences were classified as lineage 5 (L5A), except for two that were categorized as Not Determined. Sequences in Cluster III corresponded to lineages 4, 8 (L8C), and Not Determined, along with additional representation from lineage 1 (L1D, L1A, and L1E) and Lineage 11. Sequences in Cluster IV were primarily classified as lineage 1 (mainly L1B, with minor representation of L1A and L1E), as well as lineages 2 and 8 (L8C), and Not Determined. Of the two sequences in Cluster V, one was classified as lineage 5 (L5A) and the other as Not Determined. The phylogenetic tree revealed a genetically heterogeneous boundary between Clusters III and IV, where multiple lineages and sub-lineages coexisted and overlapped.

#### 3.1.2. Descriptive Epizootiology of Distribution of Clusters Across Regions in Japan (Study 1.1)

**Figure 2:**
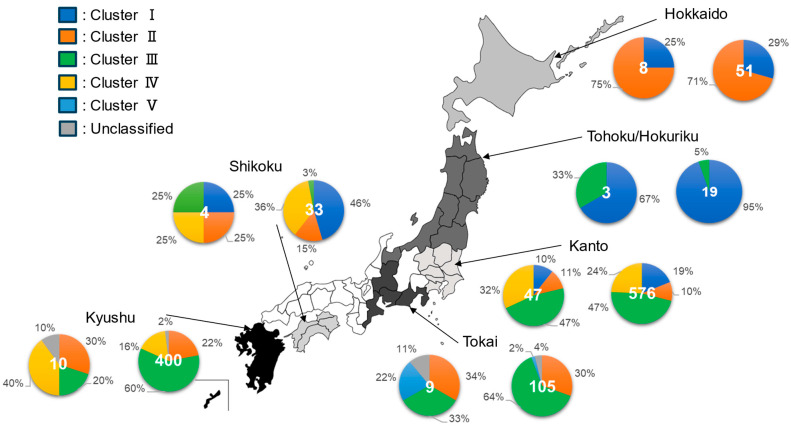
Percentage distribution of PRRSV-2 clusters across different regions of Japan, based on strains detected between 2020 and 2023. For each region shown on the map of Japan, two pie charts are presented: the distribution of clusters based on the number of farms (left) and the distribution based on the number of sequences (right). The number at the center of each pie chart represents the total count, indicating the total number of farms (left) and the total number of identified sequences (right). Clusters I to V and Unclassified strains are shown as follows: Cluster I (blue), Cluster II (orange), Cluster III (gray), Cluster IV (yellow), Cluster V (light blue), and Unclassified (green). Duplication was allowed in counting the number of farms from which multiple-cluster strains were detected. Samples from six farms of unknown location and the obtained sequences derived from them were excluded from the analysis.

In Figure 2, the regional distributions of PRRSV-2 clusters in Japan are shown. Marked differences in cluster distribution were observed among the six regions: Hokkaido, Tohoku/Hokuriku, Kanto, Tokai, Shikoku, and Kyushu. For example, only two clusters were detected in Hokkaido (Clusters I and II) and Tohoku/Hokuriku (Clusters I and III), while several clusters were detected in Kanto (Clusters I, II, III, IV), Tokai (Clusters II, III, V, Unclassified), Shikoku (Clusters I, II, IV, Unclassified) and Kyushu (Clusters II, III, IV, Unclassified). The predominant clusters by region were as follows: Cluster I in Tohoku and Shikoku (94.7% and 45.5%), Cluster II in Hokkaido (70.6%), and Cluster III in Kanto, Tokai, and Kyushu (46.7%, 63.8%, and 59.8%), respectively (Figure 2).

#### 3.1.3. Sequence Homology to the Reference Strains (Study 1.1)

Homology analysis revealed distinct sequence similarities among the PRRSV-2 clusters identified in this study. PRRSV-2 sequences belonging to Cluster I showed the highest intra-cluster homology, with a mean of 98.3% and a median of 98.3% [IQR: 97.5–99.2], and showed strong similarity to the reference strain Kyoto_93, with a mean of 93.3% and a median of 93.5% [IQR: 92.9–93.7]. Notably, sequences in Cluster I showed very high similarity to the MLV strain Fostera^®^ PRRS, with a median of 99.2% and a range of 95.2–100%. PRRSV-2 sequences belonging to Cluster II exhibited high intra-cluster homogeneity (mean: 97.8%, median: 98.5% [IQR: 97.5–99.5]) and comparable similarity to its reference strain Jam2 (mean: 93.5%, median: 94.0% [IQR: 93.7–94.2]). Sequences in Cluster II exhibited high similarity to the MLV strain Ingelvac^®^ PRRS MLV, with a median of 99.6% and a range of 88.1–100%. On the other hand, PRRSV-2 sequences in Cluster III, compared with the sequence of EDRD-1, had greater genetic variability, with lower intra-cluster homology (mean: 91.9%, median: 90.3% [IQR: 89.0–93.5]) and reduced similarity to the reference strain EDRD-1 (mean: 88.0%, median: 88.2% [IQR: 87.5–88.6]). PRRSV-2 sequences in Cluster IV, when compared with the sequence of Jpn5-37, showed the most pronounced divergence, with intra-cluster homology of 88.9% (median: 88.5% [IQR: 83.1–95.0]) and low similarity to the reference strain Jpn5-37 (mean: 84.4%, median: 84.8% [IQR: 84.0–85.1]). Meanwhile, PRRSV-2 sequences in Cluster V, compared with the sequence of Jos1, had a homology range of 82.7–88.5%, with a median of 85.6%, a 25th percentile of 84.1%, and a 75th percentile of 87.0% (Table 3). Notably, Cluster I sequences in this study showed significantly higher homology to Fostera^®^ PRRS compared with Cluster I sequences detected during 1992–2008 (92.5% [22,23], *p* < 0.05). Conversely, Cluster III sequences showed significantly lower homology to EDRD-1 than those detected during 1992–1993 (95.7% [22], *p* < 0.05).

### 3.2. Study 1.2: Regional Analysis of PRRSV-2 in the Kanto Region (2020–2023)

#### 3.2.1. PRRSV-2 Detection Rates Across Different Production Stages (Study 1.2)

PRRSV-2 was detected by RT-PCR across all developmental stages of swine production on farms in the Kanto region. The lowest positivity rate of PRRSV-2 (PCR-positive samples/total test samples) was observed in sows (28.1%) and the highest in fetuses (90.0%) (Table 4).

#### 3.2.2. Distribution of PRRSV-2 Clusters Across Production Stages (Study 1.2)

Although multiple PRRSV-2 sequences in different clusters were detected across most production stages, only PRRSV-2 sequences belonging to Clusters III and IV were detected in gilts and sows. In contrast, those belonging to Clusters I and IV were detected in aborted fetuses (Table 5). No PRRSV-2 sequences belonging to Cluster V were detected in the Kanto region.

### 3.3. Study 2: Assessment of the Relationship Between MLV Use and Distribution of PRRSV-2 Clusters of Prevalent Strains in the Kanto Region

A chi-square test showed that the proportions of pig farms using MLV differed significantly among the four identified clusters (x^2^ = 255.9, df = 3, *p* < 0.001). All farms from which PRRSV-2 sequences belonging to Clusters I and II were detected had a history of MLV use (100%, Table 6). Pairwise comparisons of proportions showed significant differences in MLV usage between the following cluster pairs: Clusters I and III (*p* < 0.001), Clusters I and IV (*p* < 0.001), Clusters II and III (*p* < 0.001), Clusters II and IV (*p* < 0.001), and Clusters III and IV (*p* < 0.001) (Table 6). The proportion of farms using MLV was the third highest in Cluster III (79.5%) and the lowest in Cluster IV (19.1%).

## 4. Discussion

This study performed a comprehensive molecular phylogenetic analysis of 1190 PRRSV-2 sequences obtained from 69 farms across Japan over a four-year period (2020–2023). The findings provide valuable insights into the genetic variability of contemporary PRRSV-2 strains circulating in the country. Comparative analysis with previously published PRRSV data and this study’s results revealed that Cluster III has undergone substantial genetic differentiation over time, resulting in notable sequence divergence from EDRD-1, the domestic reference strain, when compared with strains isolated in 1992–1993 [22]. This finding suggests that Cluster III has persisted in the country for an extended period, gradually accumulating genetic changes. Furthermore, analysis of PRRSV from farms in the Kanto region revealed that Clusters I to IV were detected frequently on farms with a history of MLV use, whereas PRRSV-2 belonging to only Clusters III and IV were detected on farms without such a history. Statistically significant differences in cluster detection rates were observed between farms with and without MLV use for each cluster. This observation strongly suggests that the use of MLV may have contributed to changes in the distribution of PRRSV-2 clusters in the region. We chose to maintain the established Cluster classification as the primary framework due to its long-standing utility and consistency in Japanese regional epidemiology. For the international context, the phylogenetic analysis (Figure 1) demonstrates that, broadly, Cluster I and II largely correspond to Lineage 8 and Lineage 5, respectively, while Cluster III is mainly associated with Lineage 4.

This study confirmed that Cluster III remains the most predominant PRRSV-2 cluster in Japan at the moment and appears to have accumulated genetic variation over time. These findings are consistent with the report by Iseki et al., who identified Cluster III between 2007 and 2008 as the most frequently detected one [23]. However, our findings differ from those of Kyutoku et al., who found that Clusters II and IV were dominant in Japan between 2018 and 2020 [13]. This discrepancy may be attributable to differences in the geographic distributions of the farms sampled, as PRRSV genetic lineages have been shown to vary depending on regional and farm-specific control strategies [8,34]. The observed pattern of genetic variation in Cluster III corresponds with the findings of Iseki et al., who reported a significant reduction in the sequence homology between Cluster III strains from 1992 to 1993 and those from 2007 to 2008 [23]. In contrast, Kyutoku et al. reported no significant changes in the sequence identity among Cluster III strains detected between 2018 and 2020 [13]. Based on the present findings, we hypothesize that genetic variation in PRRSV-2 in Cluster III occurs gradually rather than rapidly, becoming apparent only over scales of approximately a decade. These observations therefore suggest that PRRSV-2 belonging to Cluster III is undergoing slow but steady genetic diversification over extended periods by repeating the multiplications in pigs.

The low mean sequence homology of Cluster IV with its reference strain, Jpn5-37 (84.4%), suggests substantial genetic divergence. This divergence was also evident in the phylogenetic tree analysis, which showed considerable branch expansion and deep internal branching within Cluster IV, indicating the presence of multiple genetically distinct sub-lineages. Notably, comparative analysis using the international lineage classification revealed that sequences assigned to Cluster IV corresponded to several distinct sub-lineages within Lineage 1, including L1A (e.g., as recently reported [35]), L1B, and L1E, and another lineage, such as Lineage 2 and 8. These findings suggest that Cluster IV comprises a heterogeneous group of strains with substantial genetic diversity, rather than a single evolutionary lineage. This heterogeneity underscores a key limitation of the cluster-based classification system in fully capturing the evolutionary complexity of such divergent groups. To improve resolution and facilitate global comparisons, a combined classification approach is proposed—integrating detailed sequence-based phylogenetic analysis and lineage-level classification alongside traditional cluster-based methods.

Analysis of trends in Cluster I revealed that the strains detected in this study and those reported prior to the introduction of Fostera^®^ PRRS formed distinct groupings on the phylogenetic tree. Their homologies with the MLV strain showed significant differences. Kyutoku et al. similarly analyzed sequence homology to MLV strains using isolates detected between 2018 and 2020 [13]. They found that strains detected after 2019 had significantly higher homology to MLV strains than those detected in 2018 and attributed this shift to the impact of Fostera^®^ PRRS, which was introduced in 2018. Based on these results, we conclude that the detection rate of Cluster I field strains, previously distributed mainly in western Japan, has decreased in recent years. This decline likely reflects selective pressure exerted by vaccine-induced immunity—particularly against field strains closely related to the MLV strain—as well as improved sanitary management practices on pig farms, and other factors that contribute to the reduction in field strain circulation.

Our results suggest an association between MLV usage patterns and the genetic and phylogenetic distribution of PRRSV within farms. In the Kanto region, Clusters I and II were detected exclusively on the farms with a history of MLV use. Notably, many of the strains within these clusters exhibited high sequence homology to MLV strains, with mean values of 98% for both clusters. These findings suggest a strong link between MLV use and the epizootiological dynamics of Clusters I and II. This observation is consistent with the report by Kyutoku et al., who found that PRRSV Clusters I and II sequences detected between 2018 and 2020 in Japan were highly homologous to MLV strains used in Japan [13]. Similar trends have been observed in other countries, where studies have shown that the detection rate of field strains with more than 98% homology to MLV strains and decreased detection rates of strains with less than 95% homology following the introduction of MLV to the market [8,36]. Paploski et al. also noted that PRRSV genetic lineages may vary selectively according to the immune status of swine populations [21], suggesting that immunological differences between farms with and without a history of MLV use may contribute to the observed differences in the prevalence of Clusters III and IV. Although the findings indicate a strong association between MLV use and the detection of Clusters I and II, clinical data were not consistently available for the sampled farms. Consequently, the possibility cannot be excluded that these clusters represent residual, non-pathogenic vaccine-derived viruses detected following recent vaccination, rather than pathogenic field strains. Despite this limitation, the high sequence homology observed between Clusters I and II and the vaccine strains offers important insight into the potential role of MLV in shaping the genetic composition of PRRSV within farms. Taken together, our findings, which include the exclusive detection of Clusters I and II on vaccinated farms and their high sequence homology to vaccine strains, indicate that these clusters likely represent viruses of vaccine origin. The observed association between cluster distributions and farm-level MLV use history, combined with high levels of sequence homology, raises the possibility that the MLV strains persist within farms and undergo genetic changes over time. These dynamics have lasting effects on PRRSV genetic variability and the composition of prevalent strains within farms. This has important implications for the efficacy of vaccination programs and highlights the need for careful selection of vaccine strains and the potential development of new control strategies.

An important observation in this study was that although multiple PRRSV-2 clusters were detected in piglets, growers, and finishers, RT-PCR positivity was low in sows, and Clusters I and II were not detected in this age group. Several biological factors may explain this result. First, resistance to PRRSV infection is known to increase with age, and sows have physiologically robust immune defenses compared with younger pigs [37]. Second, sow herds on PRRS-positive farms are likely to have experienced repeated infections, resulting in acquired immunity to circulating field strains [38]. Third, on farms using MLV, sows are typically vaccinated at regular intervals, which may enhance specific and robust immune responses against Clusters I and II, thereby reducing the likelihood of viral replication and detection in this group [39,40]. The combined effects of these factors may have led to the lower detection rates for Clusters I and II in sows. However, this interpretation remains speculative, and additional investigations such as longitudinal sampling of individual animals and integration with serological tests are needed to better understand the dynamics of the virus in sow herds.

This study has several methodological limitations. First, sampling bias may have influenced the results due to the limited number of farms surveyed and the unequal number of sequences obtained from each farm, and the lack of detailed clinical information at the individual or farm level. This sampling bias may have disproportionately reflected the characteristics of specific regions or farm management systems, thereby limiting the generalizability of the results. Second, the analysis in this study was based solely on ORF5 sequences. Although ORF5 is commonly used for PRRSV classification, more comprehensive, whole-genome sequencing may yield more comprehensive and potentially divergent results. Third, the validity of treating populations as single clusters remains questionable, particularly for populations that exhibit substantial internal variability, such as Clusters III and IV. These limitations constrain the interpretation and generalization of the results and highlight challenges that should be addressed in future research. Subsequent studies would benefit from sampling more farms using statistically appropriate sample sizes, incorporating whole-genome sequencing to capture PRRSV genetic diversity more comprehensively, and adopting alternative taxonomic frameworks, such as lineage classification. Furthermore, adopting a longitudinal sampling design would enable the monitoring of within-farm viral dynamics over time and provide a more accurate assessment of the long-term impact of vaccine use.

## 5. Conclusions

This study highlighted the genetic diversity and evolutionary dynamics of PRRSV-2 in Japan, confirming Cluster III as the predominant cluster exhibiting gradual changes in genetic variation over time. The use of MLV may influence cluster distribution, with Clusters I and II primarily detected on farms that have a history of MLV use. Additionally, Cluster IV exhibited substantial genetic divergence, suggesting the presence of multiple distinct sub-clusters within this group. Despite methodological limitations, this study emphasized the importance of continued molecular surveillance and the refinement of classification frameworks. Future research should incorporate whole-genome sequence analysis and longitudinal sampling to more comprehensively characterize PRRSV evolution and vaccine-associated dynamics, thereby contributing to the development of improved disease control strategies.

## Figures and Tables

**Table 1:** Reference sequences of Japanese-origin PRRSV-2 strains used for phylogenetic analysis and their cluster classification.

Phylogenetic Cluster	Year of Isolation	Prefecture	Name of Isolate	Accession No.
I	1993	Aichi	Aichi N20	AB175715
I	1993	Kyoto	Kyoto 93 *	AB175724
I	1993	Nagasaki	Nagasaki 93	AB175725
I	1993	Kagoshima	Kagoshima N14	AB175723
I	2000	Ishikawa	Jis1	AB175694
I	2000	Ishikawa	Jis2	AB175695
I	2000	Shiga	Jsi1	AB175701
I	2000	Yamaguchi	Jyc1	AB175709
II	2000	Aomori	Jam2 *	AB175690
II	2000	Yamagata	Jyt2	AB175713
II	2007–2008 **	Miyagi	Miyagi08-1	AB546104
II	2007–2008 **	Osaka	Osaka08-1	AB546120
II	2007–2008 **	Osaka	Osaka08-2	AB546121
III	1992	Chiba	EDRD-1 *	D45852
III	1992	Gunma	Gu922M	AB175721
III	1993	Hokkaido	EDRD-8	AB175720
III	1993	Aomori	Aomori93	AB175716
III	1993	Ibaraki	Ibaraki3	AB175722
III	1993	Chiba	Chiba 14	AB175717
III	1993	Tokyo	Kitasato 93-1	AB023782
III	2000	Iwate	Jiw1	AB175696
III	2000	Iwate	Jiw2	AB175697
III	2000	Yamagata	Jyt1	AB175712
III	2000	Yamagata	Jyt3	AB175714
III	2000	Niigata	Jnt1	AB175698
III	2000	Ibaraki	Jib1	AB175693
III	2000	Tochigi	Jtg1	AB175708
III	2000	Saitama	Jst1	AB175702
III	2000	Yamanashi	Jyn1	AB175710
III	2000	Shizuoka	Jsz2	AB175704
III	2000	Ehime	Jeh1	AB175691
III	2001	Saga	Jsa1	AB175700
III	2007–2008 **	Aomori	Aomori08-1	AB546102
III	2007–2008 **	Miyagi	Miyagi08-2	AB546105
III	2007–2008 **	Miyagi	Miyagi08-3	AB546106
III	2007–2008 **	Yamagata	Yamagata08-1	AB546108
III	2007–2008 **	Ibaraki	Ibaraki08-1	AB546109
III	2007–2008 **	Ibaraki	Ibaraki08-2	AB546110
III	2007–2008 **	Ibaraki	Ibaraki08-3	AB546111
III	2007–2008 **	Ibaraki	Ibaraki08-4	AB546112
III	2007–2008 **	Ibaraki	Ibaraki08-5	AB546113
III	2007–2008 **	Ibaraki	Ibaraki08-6	AB546114
III	2007–2008 **	Shizuoka	Shizuoka08-1	AB546118
III	2007–2008 **	Kochi	Kochi08-1	AB546123
IV	2008	-	Jpn5-37 *	AB546125
IV	2020	-	6145-1L	LC741368
III	2007–2008 **	Saga	Saga08-1	AB546124
V	2000	Osaka	Jos1 *	AB175699

* Reference strains used for homology analysis in this study, ** The exact obtained year is not provided in the annotation.

**Table 2:** Reference sequences of non-Japanese-origin PRRSV strains used for phylogenetic analysis and their cluster classification.

Phylogenetic Cluster	Year of Isolation	Country/ Region	Name of Isolate	Accession No.
I	2006	China	JXA1	EF112445
I	2019 *	USA	Fostera^®^ PRRS	MK820650
II	1992	USA	VR2332	EF536003
II	1997 *	USA	Ingelvac^®^ PRRS MLV	AF020048
II	2000	China	BJ-4	AF331831
II	2002	South Korea	CNA18	DQ473472
II	2005	Taiwan	ML2	EU273672
II	2006	Taiwan	YL8	EU273700
IV	1994	Canada	IAF-EXP91	L40898
IV	1994	Canada	Quebec 807/94	Z82995
IV	2001	USA	MN184A	DQ176019
IV	2006	USA	PRRSV0000008558	EU758599
V	2006	South Korea	LMY	DQ473474
PRRSV-1	2019	Spain	PRRSV1-Fag	MZ318699

* Year of submission to GenBank.

**Table 3:** Nucleotide sequence homology of PRRSV-2 ORF5 genes obtained between 2020 and 2023, compared with reference strains.

Cluster	No. of Obtained Sequences	Reference Strain * (Year of Isolation)	Intra-Cluster Homology (Mean/Median [IQR])	Homology to Reference Strain (Mean/Median [IQR])
I	158	Kyoto_93 (1993)	98.3/98.3 [97.5–99.2]	93.3/93.5 [92.9–93.7]
II	221	Jam2 (2000)	97.8/98.5 [97.5–99.5]	93.5/94.0 [93.7–94.2]
III	577	EDRD-1 (1992)	91.9/90.3 [89.0–93.5]	88.0/88.2 [87.5–88.6]
IV	219	Jpn5-37 (2008)	88.9/88.5 [83.1–95.0]	84.4/84.8 [84.0–85.1]
V	2	Jos1 (2000)		85.6/85.6 [84.1–87.0]
Unclassified	13			
Total	1190			

* The earliest reference strain identified within the cluster.

**Table 4:** Positivity rates of PRRSV-2 RT-PCR by production stage on pig farms in the Kanto region.

	Pig Production Stage						
	Gilt	Sow	Fetus	Piglet	Grower	Finisher	Unknown
No. of positive samples /No. of samples	35/61	70/249	9/10	49/116	601/927	85/262	27/45
Positivity rate (%)	57.4%	28.1%	90.0%	42.2%	64.8%	32.4%	60.0%

**Table 5:** Distribution of PRRSV-2 phylogenetic clusters by production stage on farms in the Kanto region.

	Pig Production Stage													
Cluster	Gilt (%) (n/N)		Sow (%) (n/N)		Fetus (%) (n/N)		Piglet (%) (n/N)		Grower (%) (n/N)		Finisher (%) (n/N)		Unknown (%) (n/N)	
I	0.0%	(0/7)	0.0%	(0/23)	33.3%	(1/3)	3.6%	(1/28)	17.9%	(82/459)	50.0%	(17/34)	36.4%	(8/22)
II	0.0%	(0/7)	0.0%	(0/23)	0.0%	(0/3)	10.7%	(3/28)	11.5%	(53/459)	2.9%	(1/34)	4.6%	(1/22)
III	28.6%	(2/7)	17.4%	(4/23)	0.0%	(0/3)	35.7%	(10/28)	52.3%	(240/459)	11.8%	(4/34)	40.9%	(9/22)
IV	71.4%	(5/7)	82.6%	(19/23)	66.7%	(2/3)	50.0%	(14/28)	18.3%	(84/459)	35.3%	(12/34)	18.2%	(4/22)
V	0.0%	(0/0)	0.0%	(0/0)	0.0%	(0/0)	0.0%	(0/0)	0.0%	(0/0)	0.0%	(0/0)	0.0%	(0/0)

Values are expressed as percentages. **N** indicates the total number of sequences obtained for each production stage, and **n** denotes the number of sequences classified into each phylogenetic cluster within the corresponding stage.

**Table 6:** Comparisons of MLV vaccine usage among farms by the PRRSV-2 cluster in the Kanto region.

Cluster	Sequences from Farms Using MLV	Sequences from Farms Not Using MLV	Sequences from Farms with Unknown MLV Status	Total
I	109	0	0	109
II	58	0	0	58
III	213	55	1	269
IV	26	110	4	140
Total	406	165	5	576

## Data Availability

The data presented in this study are available on request from the corresponding author.

## Abbreviations

The following abbreviations are used in this manuscript:

GP	glycoprotein
MLV	modified live vaccine
NIBS	Nippon Institute of Biological Science
ORF	open reading frame
PRRS	porcine reproductive and respiratory syndrome
PRRSV	porcine reproductive and respiratory syndrome virus
RFLP	restriction fragment length polymorphism
RT-PCR	qualitative reverse transcription polymerase chain reaction
RT-qPCR	quantitative reverse transcription polymerase chain reaction
